# Clinical outcomes and challenges of surgical extirpation for carotid body paraganglioma in South Korea: a single-center retrospective study

**DOI:** 10.1186/s12957-024-03390-w

**Published:** 2024-04-25

**Authors:** Jiyoung Shin, Ji-sup Yun, Young-Wook Kim

**Affiliations:** 1 Vascular Surgery, Department of Surgery, Seoul National University College of Medicine, Seoul, Republic of Korea; 2 Department of Surgery, Kangbuk Samsung Hospital, Sungkyunkwan University School of Medicine, Seoul, Republic of Korea; 3 Vascular Surgery, Department of Surgery, Incheon Sejong Hospital, 20, Gyeyangmunhwa-ro, Gyeyang-gu, Incheon, Republic of Korea

**Keywords:** Carotid body paragangliomas, Cranial nerve injuries, Treatment outcome

## Abstract

**Background:**

Carotid body paraganglioma (CBP) is a rare, highly vascularized, and slow-growing neuroendocrine tumor. Surgical resection is the definitive treatment for CBP, however, it remains challenging due to the tumor’s proximity to critical blood vessels and cervical cranial nerves. This study aimed to document the characteristics of CBP and examine the clinical outcomes of patients following surgical extirpation of CBP.

**Methods:**

This is a single-center retrospective review analyzed patients who underwent CBP extirpation. We examined the patient demographics, preoperative clinical features, tumor characteristics, levels of catecholamines and their metabolites in the serum and urine. Surgeries were performed by one vascular surgeon with follow-ups at 1,3,6 months and yearly thereafter. Logistic regression analysis was conducted to identify risk factors associated with the occurrence of either permanent or temporary cervival cranial nerve palsy (CNP).

**Results:**

From September 2020 to February 2023, this study examined 21 cases of CBP removal surgeries that were carried out in 19 patients. The mean age of the patients was 38.9 ± 10.9 years and the percentage of males was 57.1% (*n* = 12). The most common preoperative clinical feature was painless neck mass (*n* = 12; 57.1%). Complete resection was achieved in 20 cases; excluding one case with pathologically proven sclerosing paraganglioma. Vascular procedures were performed in four cases (ECA resection, *n* = 2; primary repair of ICA tear without carotid shunting, *n* = 1; and ICA patch angioplasty with carotid shunting, *n* = 1). Temporary cranial neurologic complications, specifically aspiration and hoarseness occurred in four (19.0%), and three (14.3%) cases, respectively. Hoarseness associated with permanent CNP persisted for more than 6 months in two cases (9.5%). No recurrence or mortality was observed during the follow-up period.

**Conclusions:**

Surgical resection is the primay treatment approach for CBP; however, it poses risks of vascular or cervical CNP. The intraoperative estimated blood loss was the only identified risk factor for CNP.

## Background

We previously described carotid body paraganglioma (CBP) characteristics different from cervical schwannoma [1]. In 2017, the World Health Organization (WHO) published guidelines regarding adrenal and extra-adrenal paraganglioma [2]. Paraganglioma was previously reported that it has family history, distant metastasis, tumor recurrence, extra-adrenal location, and occurrence in children; the incidence of 10% in all of these respectively. The current diagnostic technological techniques and understanding of this tumor have shown that the frequencies of distant metastasis, family history, and extra-adrenal location were higher than the previously reported frequencies [3]. Moreover, the prevalence of altered germline susceptibility of the tumor was estimated to be 30–40%, and mainly involved genes encoding succinate dehydrogenase (SDHx) subunits. *SDHB* mutation is the underlying cause of paraganglioma development in 50–97% of the cases resulting from decreased SDH production, consequent carotid body hypoxia, hyperplasia, and tumor development [4].

Although CBP is the most common primary tumor derived from paraganglionic tissue at the carotid artery bifurcation, it is a rare tumor with incidence of 1–2/100,000, accounting for < 0.5% of all human tumors. According to the 4th edition of the WHO, paragangliomas are no longer classified as benign and malignant, as any lesion has a metastatic potential and there are no specific features that can predict the metastatic behavior. Moreover, some tumors are lethal without metastatic spread, by nature of local invasion involving critical structures [5]. Furthermore, the identification of metastases is complex, particularly in patients with germline predisposition syndromes, since multiple lesions may represent multifocal primary tumors rather than metastatic spread. Identification of paragangliomas in unusual locations such as lung or liver is not diagnostic of metastasis, since these may be primary sites. Despite of its metastatic property of these tumor, surgical resection still remains the definitive primary treatment [6]. However, surgery may be challenging due to tumor invasion to the carotid artery and cervical cranial nerves (CN). Moreover due to its rarity of CBPs, it is hard to get sufficient experiences of treatment of CBP. In this study, we aimed to report our surgical experience of CBP patients in a changed management guidelines.

## Methods

### Patients

This study protocol was approved by the Institutional Review Board of Kangbuk Samsung Hospital (IRB number: 2023-05-044). This retrospective study was conducted using a database of all consecutive patients who underwent extirpation of CBP between September 2020 and February 2023 in the Department of Vascular Surgery, Kangbuk Samsung Hospital. The following data were extracted from the medical records of each patient: demographics, preoperative symptoms, imaging and laboratory studies, surgical information, confirmed pathologic information and Grading of Adrenal Pheochromocytoma and Paragangliomas (GAPP) scores, and postoperative complications. Given the retrospective nature of this study, the requirement for informed consent was waived.

### Preoperative evaluations

We used contrast-enhanced computed tomography (CT) or magnetic resonance imaging of the head and neck as a preoperative imaging study. Functional imaging using metaiodobenzylguanidine (MIBG), Fluorodopa, or ^68^Ga-DOTATATE-positron emission tomography-computed tomography (PET-CT) was performed in patients with family history of CBPs, bilateral tumor, or patient aged < 30 years of age. In addition to the routine preoperative laboratory evaluation, we measured the preoperative serum levels of epinephrine, norepinephrine, dopamine, and their metabolites; metanephrine, normetanephrine, and vanillylmandelic acid (VMA) using liquid chromatography after 6-hour fasting and 20-min resting. Moreover, we measured 24-hour urine epinephrine, norepinephrine, dopamine, and their metabolites. These measurements were performed to rule out functional CBPs and determine the GAPP score to predict the potential risk of tumor metastasis. Functional CBPs were defined when serum and urine catecholamines and/or their metabolites showed two-fold higher levels of upper nornmal range [7]. Preoperative alpha blockers were administered for two weeks or longer in the patients with elevated catecholamine levels.

For genetic analyses of SDH subunit mutation, *SDHB* and *SDHD* were performed for patients with family history or bilateral CBPs. Tumor embolization was not performed preoperatively.

### Surgical technique

Ultrasonographic skin mapping was routinely performed preoperatively to acquire anatomical information of the tumor and carotid arteries. All surgeries were performed by one senior vascular surgeon. Each patient was positioned in neck extension to undergo CBP surgical extirpation and turned to the opposite side under general anesthesia. Upper cervical transverse skin incisions were made for small-sized tumors and oblique skin incisions along the anterior border of the sternocleidomastoid muscle were made for large-size tumors. Bipolar electrocautery was used during the tumor dissection to reduce the risk of cervical CN injury. To reduce bleeding during the tumor resection, we attempted to make 6 − 0 prolene suture ligations of the feeding branches of the tumor that are directly originating from the external carotid and carotid bifurcation. Carotid shunting was performed using a Pruitt F3 carotid Shunt (LeMaitre Vascular, Burlington, MA) under systemic heparinization in the patient who experienced carotid artery tear during the tumor extirpation. Afterwards, the bleeding was securely controlled by primary repair or patch angioplasty using bovine pericardial patch. For bilateral cases, the CBPs were removed in staged operations and thorough examinations were carried out for possible distant metastases.

### Pathologic examination and follow up

All tumor specimens were sent to the pathology department for histological confirmation and assessment of metastatic risk. Immunohistochemistry for CK (AE1/AE3), CD56, Ki-67, Synaptophysin, Chromogranin A, and S100 was performed to distinguish CBP from other neuroendocrine neoplasms. The GAPP score was used to assess the tumor metastatic risk.

Postoperatively, neurologic examinations were routinely performed for all patients to detect cervical CNP, such as the hypoglossal, vagal, or mandibular branch of the facial nerve and sympathetic trunk. Follow-up visits were conducted 1, 3, and 6 months and 1 year postoperatively. From one year postoperatively, whole body functional scan using MIBG or DOTA-(Tyr3)-octritide (DOTATOC) was performed annually to evaluate for recurrence or distant metastasis of CBPs.

### Statistical analysis

Categorical variables are expressed as numbers and percentages (%), while continuous variables are presented as mean ± standard deviation. Logistic regression analysis was performed to identify the risk factor significantly associated with either permanent or transient. Due to the small number of patients in this study, multivariable logistic regression analysis was conducted on the suspected variables collectively. All analyses were conducted using IBM SPSS Statistics for Windows, version 28 (IBM Corp., Armonk, N.Y., USA).

## Results

From September 2020 to February 2023, consecutive 21 cases of surgical removal of CBP were performed in 19 patients. Patient demographics and tumor characteristics are summarized in Table 1. All patients were of Korean ethnicity. The mean age of the patients was 38.9 ± 10.9 years, and it was more common in males (57.1%) than in females. The most common clinical presentation was palpable painless swelling in the submandibular triangle of the neck (57.1%), followed by asymptomatic, non-palpable cervical mass detected at regular health checkups (33.3%). Only two CBPs (9.6%) presented with clinical symptoms such as tongue deviation or syncope. Five (23.8%) tumors were bilateral which developed in three (15.8%) patients. One patient was referred to us after removal of opposite site glomus tumor jugulare at another hospital, which resulted in permanent vocal cord palsy. We conducted CBP surgery with a preemptive temporary tracheostomy instead of endotracheal intubation during general anesthesia.

**Table 1:** Patient demographics and tumor characteristics

Characteristics	*N* = 21 (%)
Number of tumors	21
Number of patients	19
Age (mean ± SD), years	38.9 ± 10.9
Male : female	12 (57.1%) : 9 (42.9%)
Preoperative clinical feature Painless neck mass Detected on health screening Tongue deviation Episodes of syncope	12 (57.1%) 7 (33.3%) 1 (4.8%) 1 (4.8%)
Location Left Right Bilateral	9 (42.9%) 7 (33.3%) 5 (23.8%)
Tumor size (maximal diameter on CT image, mm)	36.1 ± 12.9
Shamblin class Class I Class II Class III	3 (14.3%) 9 (42.9%) 9 (42.9%)
History of hypertension	3 (14.3%)
Preoperative catecholamine levels (mean ± SD)* Serum epinephrine (µg/mL) Serum norepinephrine (pg/mL)	48.8 ± 57.0 370.1 ± 218.9
Preoperative catecholamine metabolite levels * Serum metanepinephrine (nmol/L) Serum normetanepinephrine (nmol/L)	0.2 ± 0.1 0.5 ± 0.2
Preoperative 24-h urine catecholamine metabolites Metanepinephrine (µg) Normetanepinephrine (µg) Dopamine (µg) Vanillylmandelic acid (VMA) (µg)	116.5 ± 44.2 273.6 ± 137.6 341.1 ± 118.9 4.5 ± 1.4
Distant metastasis Breast, spine, and lung	2 (9.5%) †

*Serum preoperative catecholamine and catecholamine metabolite levels were measured using a liquid chromatography technique immediately after the 6-h fasting and 20-min resting

†Two cases (bilateral) for one patient

CT, computed tomography; SD, standard deviation

We classified the level of the tumor based on the distance from the upper margin of the second cervical vertebral body. The upper margin of the tumor extended to above C2 (*n* = 3, 11.1%), at C2 (*n* = 5, 18.5%), and below C2 (*n* = 13, 48.1%) respectively. The mean maximum diameter of the tumor measured on a CT image was 36.1 ± 12.9 mm. By Shamblin classification, 3 cases (14.3%) were class I, 9 cases (42.9%) were class II, and 9 cases (42.9%) were class III, respectively. Three patients were hypertensive taking antihypertensive medication preoperatively.

**Fig. 1:**
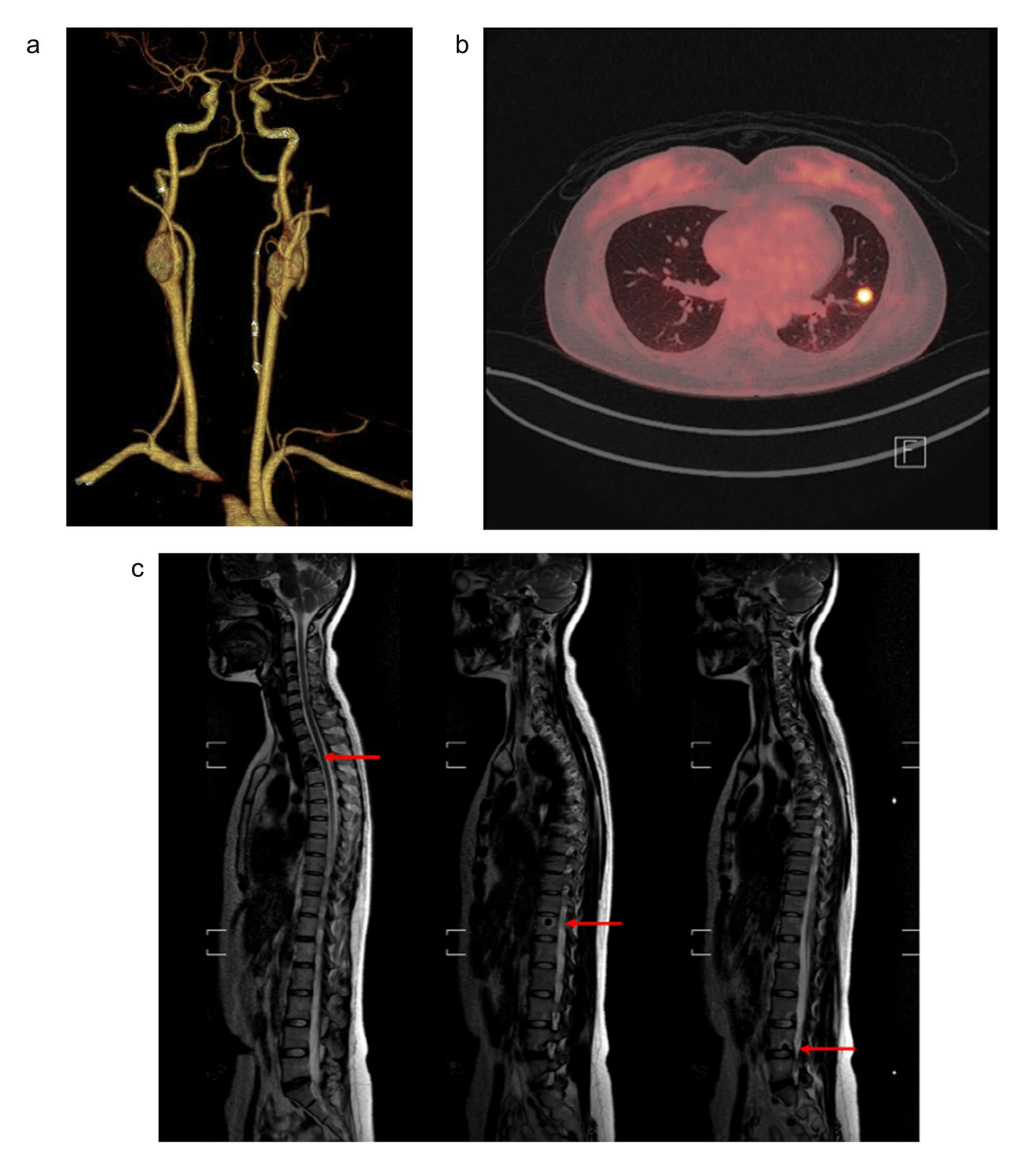
Bilateral carotid body paragangliomas (CBPs) with breast, spine, and lune metastasis (**a**) Head and neck computed tomography angiography reveals 26.4 × 16.5 mm and 25.2 × 17.3 mm sized right and left CBPs, respectively. (**b**) The large hyper-enhancing solid nodule at the left lower lobe of the left lung (12 mm) on positron emission tomography computed tomography presenting as metastasis of paraganglioma (**c**} Several different-sized sclerotic lesions in the thoracolumbar spine on spinal magnetic resonance imaging presenting as metastasis of paraganglioma

In endocrine studies, no significant abnormalities were found from the preoperative blood and urine catecholamine and metabolite testing. Though two of the three hypertensive patients showed higher urine dopamine levels, it was not statistically significant (459.6 µg vs. 321.4 µg, *p* = 0.205). One female patient with bilateral CBP had distant metastasis to the breast, bone, and lung on a PET-CT (Fig. 1) at initial presentation. No mutations were detected in the genetic tests for *SDHB* and *SDHD* performed in patients with bilateral or metastatic disease. For patients with distant metastasis, we recommneded peptide receptor radionuclide therapy or chemotherapy after removal of the primary tumor.

**Fig. 2:**
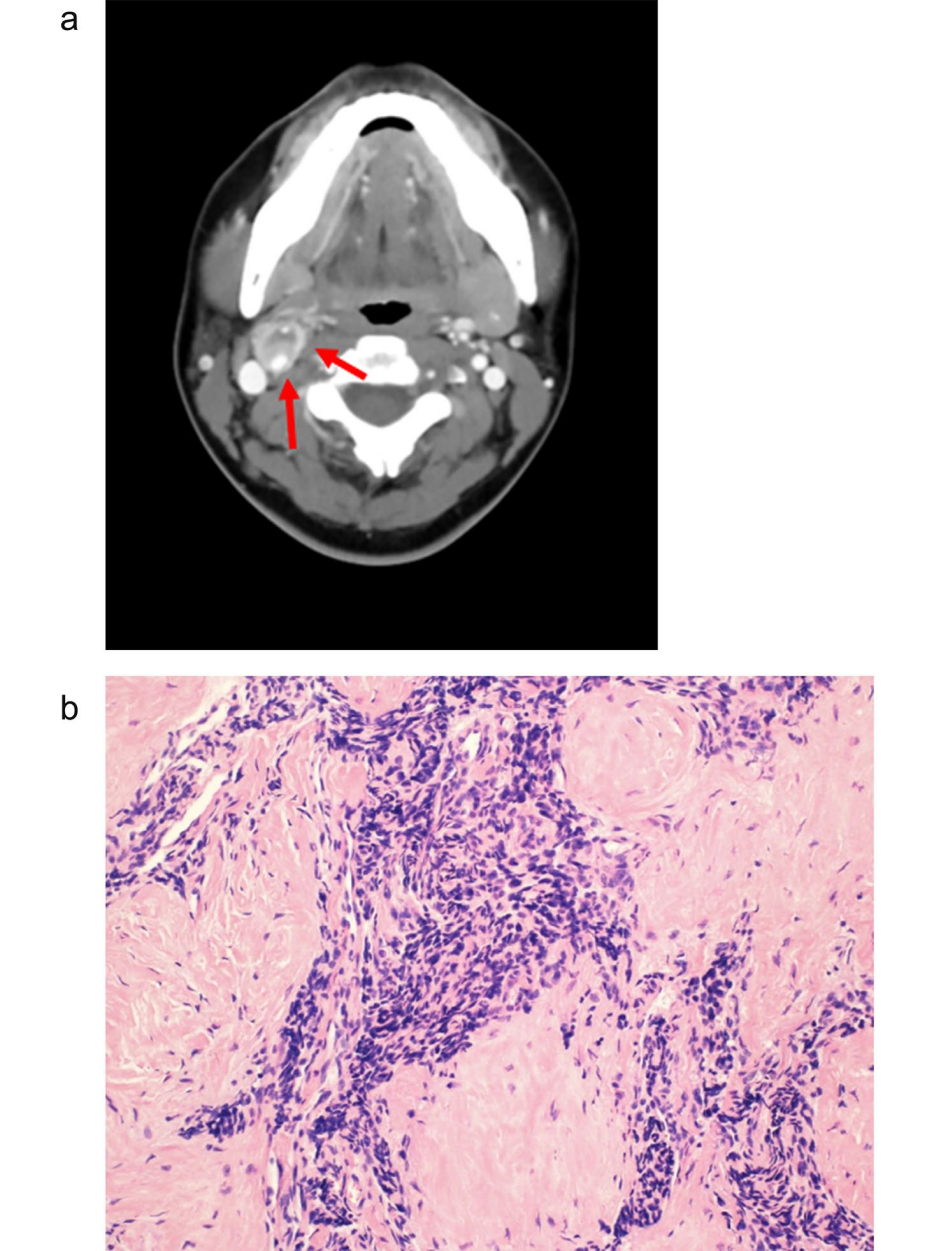
A 32-year-old female with a Shamblin III carotid body paragangioma (CBP) underwent incomplete resection. (**a**) Contrast-enhanced head and neck computed tomography scan showing the right CBP firmly attached to and surrounding the right internal carotid artery (arrow) (**b**) Histologic examination (H&E stain, 40×) of the partially excised tumor confirmed as a sclerosing paraganglioma which shows an abundant sclerotic stroma separating irregular nests and tiny bundles of the different-sized epithelioid to spindle cells is the most striking feature

The surgical details are presented in Table 2. The mean operative time and estimated blood loss (EBL) were 170.5 ± 49.8 min and 211.4 ± 193.4 mL, respectively. None of the patients required blood transfusion during or after surgery. As an adjuvant procedure, external carotid artery (ECA) resections with the tumor (*n* = 2) and internal carotid artery (ICA) repairs (*n* = 2) were required all in Shamblin III patients. Of the two ICA repairs, one case involved primary repair without carotid shunting, whereas the other case involved bovine patch repair following carotid shunt insertion due to an ICA tear. In a 32-year-old female patient with a Shamblin III tumor, complete resection was impossible without concurrent resection of the cervivcal nerve and carotid artery due to firm attach of the tumor with surrounding structure (Fig. 2-a). Histologic examination of the partially excised tumor confirmed it as a sclerosing paraganglioma (Fig. 2-b). For the patient, postoperatively, four rounds of radiation therapy (10, 24, 40, and 50 Gy in 5, 12, 20, and 25 fractions, respectively) were conducted.

**Table 2:** Treatment of carotid body paraganglioma

Variables	*N* = 21 (%)
Operative time (min)	170.5 ± 49.8
Bleeding volume (mL)	211.4 ± 193.4
Concurrent procedure ECA resection Primary repair of ICA tear without shunting ICA patch angioplasty with carotid shunting Temporary tracheotomy	2 (9.5%) 1 (4.8%) 1 (4.8%) 1* (4.8%)
Complete resection	20 † (95.2%)
Postoperative treatment Chemotherapy Radiation therapy	2 (9.5%) 1 † (4.8%)
GAPP score 0–2 3–6 7–10	3 17 1

*In a patient with permanent paralysis of the contralateral vocal cord due to previous surgery for a contralateral carotid body paraganglioma

† Incomplete resection and radiation therapy was done for a patient with sclerosing paraganglioma

GAPP, Grading System of Adrenal Pheochromocytoma and Paraganglioma; ECA, external carotid artery; ICA, internal carotid artery

Table 3 demonstrated surgical complications associated with CBP resection (Table 3). As shown in Table 3, aspiration and hoarseness were the most common complications, followed by ipsilateral marginal nerve palsy, Horner’s syndrome, and first bite syndrome. For the two patients (9.5%) with permanent vocal cord palsy, vocal cord augumentation with hyaluronic acid (Restylane, Q-Med AB; Uppsala, Sweden) injection were performed at the otolaryngology department. Follow-up was possible in all patients during the mean follow-up period of 224.7 ± 192.0 days, no tumor recurrence or new metastasis was detected during the period. To assess risk factors associated with CNP, we conducted a multivariable logistic regression analysis. On the analysis, estimated intraoperative blood loss (EBL) was the only risk factor for the nerve palsy (Table 4).

**Table 3:** Postoperative complication for carotid body paraganglioma resection

Variables	*N* = 21 (%)
Transient cranial nerve palsy Aspiration Hoarseness First bite syndrome Ipsilateral marginal mandibular nerve Horner syndrome	7 (33.3%) 4 (19.0%) 3 (14.3%) 2 (9.5%) 1 (4.8%) 1 (4.8%)
Permanent* cranial nerve palsy Hoarseness	2 (9.5%)
Tumor recurrence ** during the follow-up	none
Surgical mortality	none

* We defined permanent palsy as neurologic symptoms or signs persisting for longer than 6 months

** Tumor recurrence was determined by contrast-enhanced cervical CT and MIBG or DOTATOC RI scintiscanning

**Table 4:** Risk factor analysis predicting transient and permanant cranial nerve palsy

Variables	OR (95% CI)	*p*-value
Shamblin class Class I Class II Class III	Reference 2.50 (0.16–38.60) 0.63 (0.09–4.22)	0.512 0.630
Estimated blood loss	1.025 (1.003–1.048)	0.025
Proximal extent of the tumor Above C2 body At the C2 body Below C2 body	2.40 (0.29–19.78) 0.80 (0.06–11.29) Reference	0.416 0.869
Tumor size	1.04 (0.967–1.120)	0.292

## Discussion

Many uncertainties still exist regarding to the etiology of CBP while many hypotheses have been advocated. Since the 4th edition of the WHO classification of pheochromocytomas and paragangliomas (PPGL), PPGL is no longer classified as simply benign or malignant because any PPLG can have metastatic potential and lack clear characteristics needed to predict metastatic behavior [2]. Additionally, some tumors are fatal without metastatic proliferation due to local invasion of critical structures. Therefore, any PPGL, including CBP, should be approached as having a metastatic potential with a genetic predisposition [3]. Furthermore, PPGLs are among the most common hereditary tumors in humans, with mutation rates reaching approximately 40% and 85% in adult and pediatric patients, respectively [8, 9]. Immunohistochemical biomarkers, morphological features, and biochemical findings play a crucial role in germline susceptibility assessment [10]. The importance of the genetic study has been emphasized, particularly *SDHB* mutation examination in patients with a family history of bilateral occurrence, which may be prognostically unfavorable in head and neck locations. The 2022 WHO classification encourages the routine use of *SDHB* immunohistochemistry as a surrogate biomarker in SDHx-related pathogenesis [3].

Although active surveillance is reportedly a superior option for CBP management [11, 12], complete surgical resection is believed as a primary treatment of choice when it is available [1, 6, 13]. When considering surgical removal of CBP, we worry about two things; intraoperative bleeding and CNP. Intraoperatvie bleeding during CBP surgery is notorious due to its too many feeder vessels and risk of carotid injury. According to previous studies, 7–24% of cases required vascular reconstruction during the CBP surgery. To reduce the risk of surgical bleeding and avoid carotid injury during the surgery, preoperative tumor embolization through the feeding vessels were conducted [14, 15]. However, it carries small risk of stroke and it can extend the length of hospital stay, may trigger inflammation and lead to increased blood loss during surgery [16, 17]. In past we routinely performed preoperative tumor embolization, however we do not perform now. In our series, one patient required primary repair of the damaged ICA and another patient underwent carotid shunt insertion and angioplasty using a bovine pericardial patch. In case of severe tumor adhesion to the ECA, reconstruction of ECA is not essential after an enbloc resection of the tumor with ECA. Howeveer, ICA injury requires immediate reconstruction often using systemic heparinization, carotid artery clamping and carotid shunting and meticulous repair. Therefore, surgical team should be prepared for this event.

Second thing we have to consider is cervical CNP. It is the most common surgical complication during CBP extirpation, with a reported incidence rate of up to 50% [18]. The potential risk or nerve injury may arise from these tumors being close to the surrounding nerve structures, limited accessibility and visibility of the nerve structures during surgery due to bleeding, high location of the tumor, and distortion of normal anatomical relationships. Previous studies have tried to identify risk factors for cervical CN injury using Shamblin classification, tumor location from the the skull base, or tumor size [19, 20]. In our series, multivariable logistic regression analysis identified an estimated blood loss (EBL) as the only risk factor for cervical CNP. Amount of EBL may encompass difficulty of surgical procedure, carotid injury and large tumor mass. Even in cases of high Shamblin class or unfavorable tumor location, we have experienced that an effective feeder vessel suture ligation before tumor dissection can minimize bleeding and consequently reduce CN injury. Marginal branch of the facial nerve palsy usually occur due to traction injury and usually recovered spontaneously. The vagus and hypoglossal nerves are prone to be injured during CBP surgery [21]. Both nerves were visually assessed and preserved during surgery for all our cases. Subsequently, any related symptoms, such as aspiration and tongue deviation, were temporary. Hoarseness that lasted more than 6 month was observed in two patients (9.5%), which appeared to be due to recurrent laryngeal nerve injury. Damage to the cervical sympathetic nerve can cause Horner’s syndrome or first bite syndrome which is caused by denervation hypersensitivity [22]. First bite syndrome developed in two patients, however the pain symptom on the first bite improved within 1 month after its occurrence.

Most CBPs are first observed as a palpable mass or in routine health chekcup; therefore, patients do not usually consider them seriously. With the increased understanding of the tumor, metastatic potential and difficulty with surgery, it shoud be considered as a potentially serious condition. Since this issue is directly related to quality of life, patients should be informed about the potential risks before treatment, and a multidisciplinary follow-up approach should be considered. Daniel et al. conducted a trial of active surveillance in patients without symptoms or who did not agree to undergo surgery for CBPs and suggested that active surveillance is reasonable for CBP management. 24% of patients did not undergo surgery throughout the study. According to them, metastasis occurred in 3–6% and 15% in bilateral CBPs [11]. Additionally, if a tumor progression, cervical CNP, or vessel invasion occurs during active surveillance, the possibility of permanent damage to the nerve increases, making complete resection impossible.

Among our cohort, one patient with bilateral CBP showed multiple distant metastases. In patients with bilateral CBPs, there has been concern that bilateral carotid body resection might damage carotid body functions critical for survival; however, published results show that mortality rates do not increase due to bilateral carotid body resection despite the preexisting severe comorbidity [23]. The progressive behavior of CBPs has a diverse morphological presentation in the form of increased mitotic activity, vascular invasion, neural peripheral invasion, and cell polymorphism; however, none of these morphological features has been found to accurately predict these tumors’ behavior. Therefore, surgical extirpation remains the standard treatment for CBPs to decrease morbidity and mortality in these patients.

This study has some limitations. First, it was a retrospective study with a small sample size due to the rarity of the disease. Second, the follow-up period was short; however, 100% of the patients underwent the outpatient follow-up to detect tumor recurrence or metastasis. Though there was no recurrence during the follow-up period, long-term follow-up is required to see distant metastasis of tumor recurrence. In the furure, tumor metastasis and GAPP score will be reported.

## Conclusion

Surgical removal is the primay treatment of choice, however, it carries risk of vascular or cervical nerve injury. Among risk factors for CNP, we found that an intraoperative EBL was only risk factors.

## Data Availability

The source data for all figures included in the manuscript is stored in Mendeley Data (10.17632/3w8gy59zv8.1). Should it be permissible, the dataset generated and/or analyzed during the current research will be made available upon request from the corresponding author. Limited access to certain clinical data generated in the current study is restricted due to the absence of prior authorization for external sharing of data from research subjects without explicit consent.

## Abbreviations

CBP: carotid body paraganglioma
CN: cranial nerve
CN: cranial nerve palsy
CT: computed tomography
DOTA-(Tyr3)-octritide: DOTATOC
EBL: estimated blood loss
ECA: external carotid artery
ICA: internal carotid artery
MIBG: metaiodobenzylguanidine
PET-CT: positron emission tomography-computed tomography
PPGL: pheochromocytoma and paragangliomas
SDHx: succinate dehydrogenase
WHO: World Health Organization
